# Extracts from the Minutes of the New York Pathological Society

**Published:** 1855-09

**Authors:** 


### ECLECTIC DEPARTMENT,

### AND SPIRIT OF THE MEDICAL PERIODICAL PRESS

---

*Extracts from the Minutes of the New York Pathological Society.* Specially Reported for the New Jersey Medical Reporter, by E. Lee Jones, M. D, Secretary.

REGULAR MEETING, Feb. 28, 1855.

*Rupture of Uterus.*—Dr. Conant presented a specimen of lacerated uterus, with the following history: Eliza McGovern was taken in labor on Tuesday, Feb. 13, and at three o'clock in the afternoon the husband applied to the Demilt Dispensary for a physician. He was there informed that such cases were not provided for by the Institution. Still, to relieve his great anxiety, and by his own consent and request, a student of medicine, from a neighboring boarding-house, was called and went with him to the house, 223 E. 20th Street, and took charge of the case. After about an hour's delay, upon making an examination, this student discovered that the funis was prolapsed. He immediately informed the friends that there was a complication in the case, and that they must call the best physician that could be found, as he did not like to take the responsibility. When the husband arrived, some time in the evening, he went directly for a more experienced practitioner. Dr. Longrigg being called, expressed his belief that he should be able to deliver the woman in a very short time. He succeeded in bringing down the right foot and hand; but, failing in his efforts to deliver anything more, he called in a medical friend. After holding a consultation, Dr. L. says, "I demanded a fee, knowing that one would not be paid, and as I expected, we were discharged." Dr. L. ceased all efforts at delivering the woman at about 11 o'clock, Tuesday night, after which time the patient had no labor-pains at all, but commenced vomiting every thing taken into the stomach. She sank very rapidly, so that in two hours the friends thought her dying. The student remained by her all the time, to see the termination of the case. After the departure of Dr. L. and friend, the young gentleman went with the husband to nearly every physician's office in the vicinity, but failing to secure the services of any one, the young man was finally sent to Dr. Finnell, and the husband called on Dr. Conant, who immediately attended. Dr. F. arrived a few minutes later, about 11 o'clock on the morning of Wednesday. The patient was in a deplorable condition, the pulse being 150 per minute; the respiration about 50, and labored; the countenance having the anxious and pinched appearance peculiar to one suffering under rapid exhaustion; the abdomen was very tympanitic—in fact there was every indication of speedy dissolution. Upon further examination, Dr. C. found the right foot and leg as far as the knee, protruding through the vulva; the funis prolapsed some six inches; after which Dr. F. discovered the laceration of the womb. After a short consultation, Dr. C. proceeded to turn, and delivered the child in about half an hour, without much inconvenience to the patient, who expressed herself as decidedly relieved when the child was removed.

The child was known to be dead before delivery, as there was no pulsation in the cord.

Dr. C. continued in attendance until her death, which occurred twenty-four hours subsequently. After the child was removed, from twelve to eighteen inches of the intestine protruded into the vagina; these were returned, and the placenta was found to have passed through the rupture into the abdominal cavity.

The *post-mortem* examination was made the next day, and revealed a rupture, extending some three inches up the cervix uteri, and about the same distance down the vagina, upon the left side of the organ. The alimentary canal was excessively distended with gas. There was no sign of inflammation except upon the intestine, which was opposite the laceration.

Dr. Batchelder inquired what length of time after commencement of labor, ruptures most generally occurred. In his experience, it was usually within fifteen or eighteen hours. Dr. Finnell mentioned one case, where rupture took place three days after commencement of labor, and others within twenty hours.

Dr. Clark asked the usual seat of rupture. Dr. Finnell said, at the junction of the os uteri and vagina.

Dr. Isaacs mentioned four cases through the cervix, and one partially in body.

*Hip-Joint Disease.*—Dr. Isaacs next presented a specimen of disease of the acetabulum, head of femur, &c., in a female, aged 25. The history of this case is very imperfect. She was seized with severe pain in the hip and groin, gradually increasing, and extending to the leg and foot. After some weeks, the limb could not be moved without great suffering. In about six months after the attack, an abscess formed under Poupart's ligament. Pus continued to be discharged till the time of death. On tracing up the fistulous track of the abscess, it was found to reach upwards along the inner margin of the psoas muscle, and under the fascia iliaca, nearly to the second lumbar vertebra, but had no connection with the vertebra of this region. Tracing the fistulous track downwards, it passed over the inner portion of the brim of the pelvis, down to the acetabulum, through which there was a carious opening, of the size of a sixpence, into the cavity of the hip-joint. The whole surface of the acetabulum was greatly eroded—the head of the femur was also in the same condition, and much of its substance has been destroyed. The cavity of the joint was filled with pus, which also could freely pass through into that of the pelvis. The tendons of the rotator muscles were still adherent to their points of insertion about the neck of the femur, and were floating in an immense abscess, which existed among the glutei and hamstring muscles. The uterus contained in its cavity a large, fibrous polypus; the left ovary had been converted into a fibrous tumor, about the size of a large orange.

*Enlarged Prostate Gland.*—Dr. Isaacs next exhibited a specimen of an enormously enlarged *prostate gland* taken from a black man, about 60 years of age; the third lobe was about the size of a musket-ball, and so placed as to shut up completely the orifice of the bladder. It yielded, however, very easily to the point of the catheter, when that instrument was introduced, and probably presented no great obstacle to its passage during life.

*Cancer of Colon.*—Dr. Cochran presented a specimen of *cancer* of the *transverse colon*, taken from a woman 62 years of age. Three years ago, he was consulted for a diarrhœa, which continued for two months; on examining the abdomen, he found a hard mass about and below the umbilicus. From the existence of this mass, and the persistence of the dysentery, he was induced to infer malignant disease existed. The autopsy showed cancer, involving the colon, stomach, bladder, and peritoneum.

Regular Meeting, Feb. 14.

*Bright's Disease.*—Dr. Clark presented several bottles of urine containing a considerable amount of blood, and a small quantity of albumen; there was nothing else worthy of notice. The patient had been passing blood from the kidney for three weeks. In his opinion he was laboring under the first stage of Bright's disease; the blood being the result of functional congestion of the kidney. Another specimen was exhibited of the same color, taken from a gentleman, who, for the last two years, had passed bloody urine. On being placed under the microscope, it was found to be of a different nature. There were numerous cells visible, which seemed to be formed on variable laws; a few could be declared cancer cells; a few epithel ial cells of the bladder. A few days (three) after the present specimen was passed, the urone presented an entirely different appearance, having lost its deep red coler; the deposit, however, was found the same in character.

*Pathology of Inflammation.*—Dr. Clark then presented three specimens possessing some interest as showing modifications of the inflammatory process. The first specimen exhibited consisted of the lungs, trachea, and larynx of a child eight years of age, who died of croup. False membrane is seen extending down to the third division of the bronchial tubes; the trachea and larynx are also lined by the same tissue. On the fifth day (Sunday) after the commencement of the disease, tracheotomy was deemed necessary. The operation was accordingly performed by Dr. Buck, for whom the case is presented by Dr. Clark. From Sunday to Saturday the patient's condition was satisfactory, and hopes of recovery were entertained. On Saturday, however, she became restless, and respiration difficult, the tube being filled with false membrane. Dr. Mitchell, of Brooklyn, the patient being under his immediate care, was called during the night, aud removed the inner tube; a piece of membrane was violently ejected six feet from the patient; a few hours after, a portion six inches in length was thrown out. No improvement followed, and the patient died on Sunday. On examination by the microscope, the membrane (from the internal surface of the trachea) seemed as if newly formed, and composed of small cells, markedly granular, arranged in lines; a series of cells were connected by structural material; occasionally a little thread was actually formed. Magnified four hundred diameters they appeared one-quarter of an inch apart. A grayish exudation occurred on the surface of the wound, thirty-six hours previous to death; the croupous membrane seemed as if coming through the opening. The structure of this exudation was entirely different, there being no cells, no fibres, but numerous minute granules; the muscular fibres were also surrounded by minute granules. For six days after the operation, the deposit was diphtheritic, composed of protein compounds and fatty matter. In the same connection, Dr. Buck inquired of Dr. Clark, if the grayish exudation found occasionally on the surface of the wounds, refusing to heal kindly, was not of the same character? He (Dr. Clark) found on examination that it was of the same general nature; portions of the exudation contained minute granules, others a few fibres further in the same connection.

*Dysentery.*—Dr. Clark presented the large intestines of a patient, who died of dysentery, lasting thirty days, extensively ulcerated. Everywhere on the surface of the membrane, between the ulcerations, was a grayish exudation, which, on microscopic examination, was found to be constituted as the membranes just described, being composed of granules, and some portions containing fibres. These three exudations, Dr. Clark considered as products of the inflammatory process; there not being, in either case, sufficient inflammation, or *viability*, to produce a more highly organized tissue. Dr. Batchelder remarked that such exudations were more apt to be seen in cases of burns, and in feeble subjects.

*Aneurism of Heart.*—Dr. Clark then exhibited for Dr. Cox an uncommon form of aneurism—an *aneurism* of the *heart*, at the base of the mitral valve of the left ventricle, about the size of a pullet's egg; the sac was lined with coagulated blood; the tissue of the heart around it was fibrous. The history of the case is incomplete. No physical examination was held. She had been in bad health for about one year, being troubled with cough, accompanied with expectoration. The immediate cause of death was double pleurisy, with effusion. Dr. Clark next presented a heart, weighing 27 oz. It was taken from a person who had passed through an attack of pneumonia, and was convalescing. There was an inconsiderable charge of the aortic valves.

*Fibrous Tumors of Uterus.*—Dr. Finnell presented the uterus of a woman, far advanced in gestation, 25 years of age, containing 25 fibrous tumors, varying in size from a pea to the fist. She had been suffering for four days from cough and pain in the side. Coming out of church, she became sick and died during the night. The *post-mortem* revealed the lower portion of the right lung congested—the other portions were healthy; the fœtus was of full size. Dr. Clark considered it unusual for pregnancy to occur under such circumstances. Dr. Dalton called attention to the condition of the cervix uteri, the canal being perfectly distinct from the body of the womb. He considered it interesting, as disproving the old opinion that the canal gradually became obliterated as gestation advanced.—*N. J. Med. Reporter.*

